# 

**Published:** 1894-01

**Authors:** 


### CONTENTS:

Article I.—Inaugural Address. By L. D. Shepard, A. M., D. D. S., D. M. D. - - - - - - 385
" II.—The Rapid Advance of Dentistry. By Dr. B. Holly Smith. - - - - - - 407
" III.—Rigg's Disease—What is it, and can it be Cured? By Dr. G. A. Mills. - - - - - - 410
" IV.—Tin Foil Fillings. - - - - - - 416
" V.—The Breath. By Dr. J. Taft. - - - - 419

### COMMUNICATED.

The Missouri State Dental Association. - - - - - 424
Resolutions Adopted by the Chicago Dental Society on the Death of Dr. W. W. Allport. - - - - - - 424

### EDITORIAL, Etc.

The Journal in 1893. - - - - - - - - 425

Neuralgia. - - - - - - - - - - 426

### MONTHLY SUMMARY.

Number of Dental Colleges and Students. - - - - 428

For the Teeth—Some Excellent Rules to Follow in the Care of Them. 429

Deaths under Anæsthetics. - - - - - - 430

The Administration of Anæsthetics. - - - - - 430

The Use of Cocaine. - - - - - - - - 431

Hygiene of Hair Dressing and Barber Shops. - - - - 431

Iodide of Potash in Headache. - - - - - - - 432

Pinus Canadensis. - - - - - - - - 432

